# Riboflavin Production by Steady-State Continuous Cultures of *Hyphopichia wangnamkhiaoensis* in a Bubble Column Bioreactor

**DOI:** 10.3390/microorganisms13040817

**Published:** 2025-04-03

**Authors:** Raziel Arturo Jiménez-Nava, Griselda Ma. Chávez-Camarillo, Eliseo Cristiani-Urbina

**Affiliations:** 1 Departamento de Ingeniería Bioquímica, Escuela Nacional de Ciencias Biológicas, Instituto Politécnico Nacional, Unidad Profesional Adolfo López Mateos, Avenida Wilfrido Massieu s/n, Mexico City 07738, Mexico; rjimenezn@ipn.mx; 2 Departamento de Microbiología, Escuela Nacional de Ciencias Biológicas, Instituto Politécnico Nacional, Colonia Santo Tomás, Prolongación de Carpio y Plan de Ayala s/n, Mexico City 11340, Mexico

**Keywords:** chemostat, riboflavin, kinetics, kinetic parameters, *H. wangnamkhiaoensis*, Monod model, Luedeking–Piret model

## Abstract

Riboflavin is biosynthesized and excreted extracellularly by the novel yeast *Hyphopichia wangnamkhiaoensis*. The steady-state kinetics of cell growth, substrate consumption, and riboflavin production by *H. wangnamkhiaoensis* were studied in a chemostat continuous culture at different dilution rates. The unstructured Monod and Luedeking–Piret models were used to describe cell growth, substrate consumption, and riboflavin production, and crucial kinetic parameters were estimated. The experimental data fitted the proposed models well. The maximum specific growth rate, substrate affinity constant, maintenance energy coefficient, and maximum biomass yield values were 0.1378 h^−1^, 0.4166 g of glucose L^−1^, 0.1047 g of glucose g^−1^ of biomass h^−1^, and 0.172 g of biomass g^−1^ of glucose, respectively. The maximum yield from glucose and volumetric and specific productivities of riboflavin were 0.7487 mg of riboflavin g^−1^ of glucose, 0.5593 mg of riboflavin L^−1^ h^−1^, and 0.6547 mg of riboflavin g^−1^ of biomass h^−1^, respectively. The estimated growth-associated riboflavin production constant (4.88 mg of riboflavin g^−1^ of biomass) was much higher than the non-growth-associated riboflavin production constant (0.0022 mg of riboflavin g^−1^ of biomass h^−1^), indicating that riboflavin production by *H. wangnamkhiaoensis* is a predominantly growth-associated process. The chemostat continuous culture offers a promising strategy for efficiently and sustainably producing riboflavin using *H. wangnamkhiaoensis*.

## 1. Introduction

Riboflavin (vitamin B_2_) is among the most important vitamins because of its diverse and crucial physiological, metabolic, and health functions in living beings [1,2,3,4]. Riboflavin is a key component of the coenzymes flavin mononucleotide and flavin adenine dinucleotide, which participate as electron carriers in the reduction–oxidation (redox) reactions involved in numerous metabolic pathways, energy production, and cellular antioxidant function [5].

The world’s annual riboflavin production is over 10,000 tons, with global sales of approximately USD 452 million [6,7,8,9]. Approximately 70% of manufactured riboflavin is used as a feed additive, 20% as a food additive, and 10% in pharmaceuticals and cosmetics [2,3,6,8,10,11].

Riboflavin is currently produced industrially through fermentation using *Ashbya gossypii* and *Bacillus subtilis* [6,8]. However, the industrial production of riboflavin has some inherent limitations, such as long fermentation times (72–240 h), complex raw material composition, high cell viscosity, and challenging riboflavin purification [2,8,10,11,12,13,14]. Therefore, researchers are investigating new flavinogenic microorganisms that can grow in simple culture media and produce riboflavin in short fermentation times. Several bacteria, filamentous fungi, and yeasts, other than those mentioned above, have been found to overproduce riboflavin [2,3,6,10,14,15,16,17,18,19,20].

Yeasts have garnered significant attention as riboflavin producers because of their range of advantages, such as their capability to grow in simple and inexpensive culture media with simple rheological properties, making the downstream separation and purification processing of riboflavin less costly, more straightforward, and less technologically challenging [11,14,21]. Other advantages include their ease of cultivation on cheap renewable carbon and energy sources, high specific growth rate, ease of handling, low maintenance requirements, resistance against phage infection, safety, metabolic versatility, and ease of genetic manipulation [6,14,16,22]. In this context, the novel yeast strain *Hyphopichia wangnamkhiaoensis* has recently attracted much attention because of its capability to produce riboflavin within 18–24 h of incubation when grown in a simple culture medium [14,23].

The riboflavin biosynthetic pathway in yeasts involves seven enzyme-catalyzed steps that start with guanosine triphosphate (GTP) and ribulose 5-phosphate (Ru5P). GTP is converted to 2,5-diamino-6-ribosyl-amino-4(3*H*)-pyrimidinone 5′-phosphate (DARPP) by GTP cyclohydrolase II. DARPP is subsequently reduced and deaminated to 5-amino-6-ribityl-amino-2,4(1*H*,3*H*)-pyrimidinedione 5′-phosphate (ArPP), which is then dephosphorylated to 5-amino-6-ribityl-amino-2,4(1*H*,3*H*)-pyrimidinedione (ArP). Ru5P is converted to 3,4-dihydroxy-2-butanone-4-phosphate (DHBP) by DHBP synthase. DHBP and ArP are condensed to form 6,7-dimethyl-8-ribityllumazine (DMRL) by lumazine synthase (LS). Finally, two DMRL molecules are combined by the enzyme riboflavin synthase to form one molecule of riboflavin and one molecule of ArP, and the latter is recycled in the biosynthetic pathway [11,13,18].

Several strategies have been proposed to improve vitamin production, including optimization of culture media, fermentative process parameters, fermentation cultivation mode (batch culture, continuous culture, and fed-batch culture), mutagenesis, and metabolic engineering [13,24].

Regarding the fermentation cultivation mode, most studies on riboflavin production have been conducted in batch cultures. In contrast, to the best of our knowledge, little specialized literature referring to the production of riboflavin in continuous systems, such as those using immobilized *Candida tropicalis* cells [25], as well as the free cells of *Meyerozyma guilliermondii* [26], *A. gossypii* [27,28], and *B. subtilis* [29,30,31,32], is available.

Unlike conventional batch fermentation, in chemostat continuous cultures, the biomass, substrate, and product concentrations, as well as the growth, substrate consumption, and product production rates, remain constant after reaching a steady state. This attribute makes chemostat continuous culture a powerful tool for gaining valuable insights into the metabolism and physiology of microorganisms and for precisely characterizing the kinetic parameters of cell growth, substrate consumption, and product production by microorganisms. These insights are crucial for understanding the impacts of environmental factors on the performance of fermentation processes, as well as for predicting the dynamics and optimizing, designing, and scaling-up of fermentation processes [22,33,34,35,36,37].

Other advantages of chemostat continuous culture compared with other fermentation reaction systems include a higher product yield and productivity and increased economic feasibility, as the microbial biomass and products can be harvested and processed throughout the process [22,38]. Furthermore, in chemostat continuous cultures, the fermentation broth of microorganisms remains viable for a long time and over multiple runs [39,40]. Moreover, the growth inhibition caused by the substrate and by-products, resource consumption, waste generation, and time-consuming activities such as the cleaning, filling, and sterilization of bioreactors is reduced [22,39,41,42].

The primary aim of this work was to investigate the kinetics of cell growth, substrate consumption, and riboflavin production by *H. wangnamkhiaoensis* in chemostat continuous cultures. Furthermore, the critical kinetic parameters of cell growth, substrate consumption, and riboflavin production, such as the critical and optimal dilution rates, specific substrate consumption rate, maintenance energy coefficient, substrate affinity constant, biomass yield, as well as the yield and specific and volumetric production rate of riboflavin were estimated. These kinetic parameters are essential for bioprocess development, bioreactor design, and scale-up, and, to the best of our knowledge, they are missing from the literature on the *Hyphopichia* genus and riboflavin production.

## 2. Materials and Methods

### 2.1. Yeast Strain and Its Preservation

*H. wangnamkhiaoensis* ENCB-7, obtained from the Microbial Culture Collection of the Industrial Microbiology Laboratory of the Biological Sciences National School at the National Polytechnic Institute (ENCB-IPN, Mexico City, Mexico), was used throughout this work. This yeast strain was preserved at 4 °C on yeast nitrogen base (YNB)-glucose medium slants with the following chemical composition: 10 g L^−1^ glucose, 6.7 g L^−1^ YNB (BD Biosciences, Sparks, MD, USA), and 20 g L^−1^ bacteriological agar (BD Biosciences).

### 2.2. Culture Medium

A Raziel–Griselda–Eliseo (RGE) culture medium, which was previously optimized and proposed for riboflavin production by *H. wangnamkhiaoensis* [14], was used throughout this work for both yeast propagation and chemostat continuous culture experiments. The chemical composition of the RGE culture medium was as follows: 10 g L^−1^ glucose, 4.3037 g L^−1^ (NH_4_)_2_SO_4_, 0.625 g L^−1^ dibasic ammonium citrate, 1.0942 g L^−1^ KH_2_PO_4_, 0.275 g L^−1^ MgSO_4_·7H_2_O, 0.375 g L^−1^ NaCl, 15 mM glycine, and 0.02 mg L^−1^ biotin [14]. Glucose, biotin, and glycine were purchased from Sigma-Aldrich (St. Louis, MO, USA), and the other chemical components were obtained from JT Baker (Avantor Performance Materials, Inc., Xalostoc, Mexico State, Mexico).

To avoid the Maillard reaction, solutions of glucose, mineral salts, and glycine were sterilized separately by autoclaving at 121 °C for 15 min. Biotin solutions were sterilized by microfiltration using 0.22 μm mixed cellulose ester Millipore^®^ membranes (Merck KGaA, Darmstadt, Germany).

### 2.3. Inoculum Preparation

An inoculation loop of *H. wangnamkhiaoensis* was placed in a 250 mL Erlenmeyer flask containing 50 mL RGE culture medium. The flask was incubated in a water bath shaker (Cole-Parmer Instrument Company, Vernon Hills, IL, USA) at 120 rpm and 28 ± 2 °C for 15 h. After incubation, the 15-h-old culture broth was used as inoculum for further chemostat continuous culture experiments.

### 2.4. Bubble Column Bioreactor

**Figure 1:**
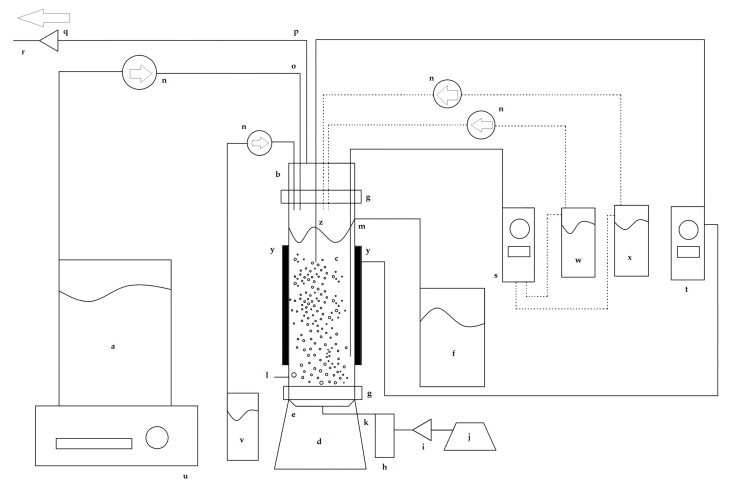
Schematic diagram of the bubble column bioreactor used for the chemostat continuous culture experiments. (a) Sterile culture medium reservoir, (b) lid, (c) cylindrical column, (d) base, (e) air diffuser, (f) effluent reservoir, (g) Nylamid flanges, (h) flowmeter, (i) air filter, (j) diaphragm pump, (k) sterile air input, (l) sampling port, (m) outflow effluent duct, (n) peristaltic pumps, (o) culture medium feed line, (p) exhaust airline, (q) air filter, (r) exit air flow, (s) pH controller, (t) temperature controller, (u) magnetic stirrer plate, (v) antifoam reservoir, (w) acid reservoir, (x) alkali reservoir, (y) heating bands, and (z) temperature sensor. The dashed lines indicate which pumps are controlled by the pH control system.

Figure 1 shows a schematic diagram of the experimental setup used in this work. The chemostat was a bubble column bioreactor with a working liquid volume of 500 mL and a total volume of 600 mL.

The base, lid, and cylindrical column of the chemostat were made of borosilicate glass. A centered porous glass diffuser in the base of the chemostat was connected to a sterile air input port. The cylindrical column had an outflow effluent duct connected to an effluent reservoir. The lid had ports for adding the sterile culture medium, antifoam, acid, and alkali, and an exhaust air outlet port. The lips of the base, column, and lid were fastened together with Nylamid flanges connected with stainless steel screws and stainless steel butterfly wing nuts. To hermetically seal the chemostat junctions and prevent glass-to-glass contact, sterile neoprene O-rings lubricated with silicone-based thermal grease were placed between the two glass lips.

A sterile RGE culture medium was fed into the chemostat at a constant flow rate using a Cole-Parmer Masterflex L/S peristaltic pump (Cole-Parmer Instrument Company). Sterile air was supplied through the porous glass diffuser at an airflow rate of 1 vvm, which was controlled using a pressure-regulating valve and measured using a calibrated rotameter.

### 2.5. Kinetics of Cell Growth, Glucose Consumption, and Riboflavin Production by H. wangnamkhiaoensis in a Chemostat

The effects of dilution rate (D) on cell growth, glucose consumption, and riboflavin production were examined in chemostat continuous cultures with a constant pH (6.0 ± 0.1), temperature (28 ± 2 °C), and sterile air flow rate (1 vvm). After inoculating the bioreactor, the process was started as batch fermentation and allowed to run for 24 h. Subsequently, the bioreactor was continuously fed with the sterile RGE culture medium at a known flow rate (F) using a peristaltic pump until the biomass, glucose, and riboflavin concentrations of the liquid effluent of the bioreactor remained constant over time (*p* > 0.05). This occurred at approximately 3–4 liquid residence times and indicated that a steady state had been reached.

Several flow rates of the RGE culture medium were assessed, thus assessing several dilution rates, which, at the steady state, are equal to the yeast-specific growth rate (μ). Culture samples were collected at a steady state and analyzed for biomass, glucose, and riboflavin concentrations.

The maximum specific growth rate of *H. wangnamkhiaoensis* was determined experimentally using the washing-out method, following the procedures described by Pirt and Callow [43]. An initial steady state was attained at a dilution rate of 0.0961 ± 0.0004 h^−1^, and then the dilution rate was suddenly increased to 0.2039 ± 0.0008 h^−1^. The decrease in biomass dry weight concentration was monitored every 15 min for 5 h.

### 2.6. Analytical Methods

The *H. wangnamkhiaoensis* biomass concentration was determined using the dry biomass weight method, as described by Chávez-Camarillo et al. [22]. For this purpose, 5 mL of culture samples were filtered through pre-weighed Whatman glass microfiber filters GF/A (1.6 μm pore size; Cytiva, Marlborough, MA, USA). The biomass retained was washed thrice with distilled water and subsequently dried at 60 °C until a constant weight was reached. Cell-free filtrates were used to determine glucose and riboflavin concentrations.

Glucose concentration was measured enzymatically using the glucose oxidase–peroxidase method [44]. The glucose content of the samples was calculated from the interpolation in a 10-point calibration curve prepared using an external glucose standard (Sigma-Aldrich).

Riboflavin concentration was measured fluorometrically using a SpectraMax M3 fluorometer (Molecular Devices LCC, San Jose, CA, USA) at an excitation wavelength of 450 nm and an emission wavelength of 525 nm [14,23]. The riboflavin content of the samples was calculated by interpolating in a 10-point calibration curve prepared with an external riboflavin standard (Supelco Inc., Bellefonte, PA, USA).

### 2.7. Determination of Kinetic Parameters

The substrate affinity constant (K_s_) of Monod’s growth model [45,46] was estimated by non-linear regression analysis of the experimental data using Wolfram Mathematica software version 10.0 (Wolfram Research, Champaign, IL, USA). The kinetic parameters of the mathematical models describing the specific substrate consumption, specific riboflavin production, and maximum specific growth rates were estimated by linear regression analysis of the experimental data using the above software.

### 2.8. Data and Statistical Analyses

Chemostat continuous cultures for riboflavin production were made at least in duplicate for each dilution rate. The measurements represent three replicates for each duplicate culture grown at the indicated dilution rates. Data are expressed as the mean ± standard deviation for the biomass, glucose, riboflavin concentrations, volumetric biomass productivity, biomass yield, specific rate of glucose consumption, riboflavin yield, specific rate of riboflavin production, and volumetric riboflavin productivity.

The statistical analysis of experimental and calculated data of biomass, glucose, riboflavin concentrations, volumetric biomass productivity, biomass yield, specific rate of glucose consumption, riboflavin yield, specific rate of riboflavin production, and volumetric riboflavin productivity was performed using one-way ANOVA followed by Bonferroni’s multiple comparison post-test (*p* < 0.05) using GraphPad Prism version 8.0.2 (GraphPad Software, Inc., San Diego, CA, USA).

## 3. Results and Discussion

### 3.1. Effect of Dilution Rate on Cell Growth and Glucose Consumption by H. wangnamkhiaoensis

**Figure 2:**
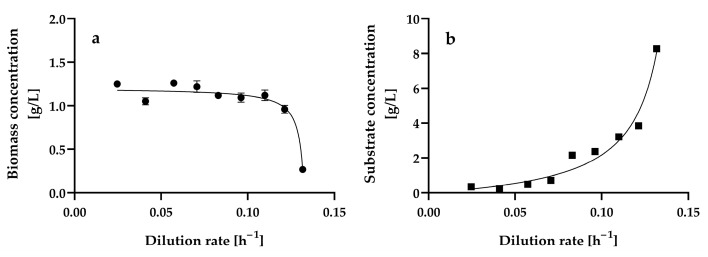
Steady-state biomass (**a**) and residual glucose (**b**) concentrations at different dilution rates in a chemostat continuous culture of *Hyphopichia wangnamkhiaoensis*. Data are expressed as mean values ± standard deviation from at least two biological replicates and three technical replicates (*n* ≥ 6). (●), biomass concentration; (■), residual glucose concentration.

Figure 2 shows the steady-state biomass and residual glucose concentrations at the different dilution rates assayed, ranging from 0.0246 ± 0.001 h^−1^ to 0.1317 ± 0.0013 h^−1^. We also attempted to assay dilution rates lower than 0.0246 h^−1^ and higher than 0.1317 h^−1^; however, in the former, a steady state could not be reached because cell agglomerates were formed, and in the latter, cell wash-out occurred.

Biomass agglomeration, wall growth, and excess foaming in the aeration bioreactor may prevent the effective mixing and liquid phase homogeneity of the bioreactor, which in turn may prevent it from reaching a steady state. In contrast, planktonic cells are suspended and move freely in liquid culture media, leading to a more uniform and homogeneous liquid phase, which is a necessary condition for the growth of microorganisms to occur at a constant rate and in a constant environment; that is, for microbial growth to take place under steady-state conditions in a continuous culture [35,47,48,49]. At dilution rates ranging from 0.0246 ± 0.001 h^−1^ to 0.1317 ± 0.0013 h^−1^, *H. wangnamkhiaoensis* grew as planktonic cells, and the steady-state condition was reached within 3–4 residence times.

The dilution rate variation produced a pattern of changes in the steady-state biomass (Figure 2a) and growth-limiting glucose concentrations (Figure 2b). This pattern is characteristic of the scenario where the biomass yield is independent of the dilution rate, and the substrate affinity constant (K_s_) (also known as the Monod, half-saturation, or half-rate saturation constant) is small relative to the inlet growth-limiting substrate concentration (S_r_) [50]. The kinetic patterns of steady-state biomass and residual glucose concentrations also suggest that the Monod model describes the growth of *H. wangnamkhiaoensis* in a chemostat [49,51,52].

The steady-state biomass concentration remained practically constant at dilution rates ranging from 0.0246 ± 0.001 h^−1^ to 0.1099 ± 0.001 h^−1^, with a mean value of 1.16 ± 0.09 g L^−1^. At later dilution rates (D > 0.1099 ± 0.001 h^−1^), the steady-state biomass concentration decreased gradually until reaching a value of 0.27 ± 0.02 g L^−1^ at a dilution rate of 0.1317 ± 0.0013 h^−1^. This suggests that this latter dilution rate (D = 0.1317 ± 0.0013 h^−1^) is close to the critical dilution rate (D_c_), which is equal to the maximum specific growth rate of a microorganism (μ_max_). In addition to the dilution rate, the steady-state biomass concentration is also dependent on the inlet growth-limiting substrate concentration [35,49].

The lowest steady-state residual glucose concentrations were reached at the lowest dilution rates assayed (Figure 2b), with no significant differences (*p* > 0.05) and a mean value of 0.35 ± 0.01 g L^−1^ in the range of dilution rates from 0.0246 ± 0.0010 h^−1^ to 0.0573 ± 0.0011 h^−1^. At dilution rates higher than 0.0573 ± 0.0011 h^−1^, the residual glucose concentration gradually increased until it reached 8.27 ± 0.09 g L^−1^ at a dilution rate of 0.1317 ± 0.0013 h^−1^. This residual glucose concentration trend was theoretically expected because, at lower dilution rates, the contact time between microbial cells and nutrients is long, and therefore, the substrate is consumed entirely or almost completely (S ≈ 0); in contrast, as the dilution rate increases, the contact time between the microorganisms and the nutrients decreases, and the microorganisms have less time to consume the substrate, resulting in increased residual substrate concentration.

**Figure 3:**
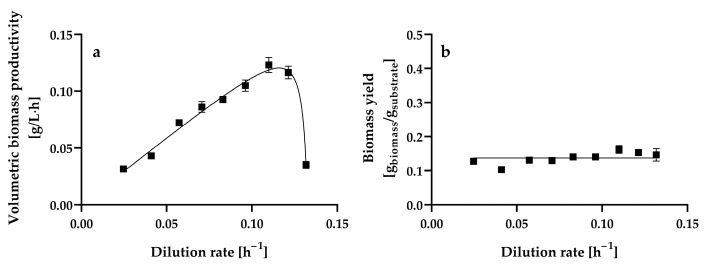
Steady-state volumetric biomass productivity (**a**) and yield (**b**) at different dilution rates by *Hyphopichia wangnamkhiaoensis* in chemostat continuous culture. Data are expressed as mean values ± standard deviation from at least two biological replicates and three technical replicates (*n* ≥ 6). (■), biomass yield.

The steady-state volumetric biomass productivity increased almost linearly as the dilution rate increased from 0.0246 ± 0.001 h^−1^ to 0.1099 ± 0.001 h^−1^, reaching a maximum value of 0.1231 ± 0.0066 g of biomass L^−1^ h^−1^, which was statistically equal (*p* > 0.05) to that obtained at a dilution rate of 0.1213 ± 0.0004 h^−1^ (0.1164 ± 0.0055 g of biomass L^−1^ h^−1^). Thereafter, the volumetric biomass productivity decreased significantly (*p* < 0.05) as the dilution rate increased from 0.1213 ± 0.0004 h^−1^ to 0.1317 ± 0.0013 h^−1^ (Figure 3a).

The steady-state biomass yield (Y) from glucose remained practically constant within the range of dilution rates assayed, with a mean value of 0.1371 ± 0.0165 g of biomass g^−1^ of glucose consumed, indicating that the biomass yield was almost independent of the dilution rate (Figure 3b). The biomass yield obtained in the present study for *H. wangnamkhiaoensis* was similar to the 0.17 [53] and 0.16 g of biomass g^−1^ of glucose [54] reported for some strains of *Saccharomyces cerevisiae* but lower than the 0.45 g of biomass g^−1^ of glucose reported for *Saccharomyces carlsbergensis* LAM 1068 [55] and the 0.51 g of biomass g^−1^ of glucose found for *Candida utilis* CBS 621 and *S. cerevisiae* CBS 8066 [56] in glucose-limited aerobic chemostat cultures.

The biomass yield of aerobic organisms depends on different factors, including the physiological and biochemical mechanisms of the organisms, cell composition, culture conditions (medium composition, type of carbon and nitrogen sources, C/N ratio, P/O ratio, temperature, pH, osmotic stress, oxygen and CO_2_ pressures, etc.), the amount of CO_2_ lost in assimilation reactions, the ATP and NADPH requirements for biomass formation, the efficiency of oxidative phosphorylation, the maintenance energy requirement, and solute transport, among others [56,57]. A high biomass yield is highly desirable when the cost of the culture medium represents a significant percentage of the cost of the final product (e.g., single-cell protein). In contrast, in some industrial processes, a low biomass yield is especially important for maximizing the conversion of the substrate to the desired extracellular product [57].

### 3.2. Determination of the H. wangnamkhiaoensis Maximum Specific Growth Rate (μ_max_), Cell Growth Model, Substrate Affinity Constant (K_s_), Maximum Biomass Yield (Y_max_), and Maintenance Energy Coefficient (m)

**Figure 4:**
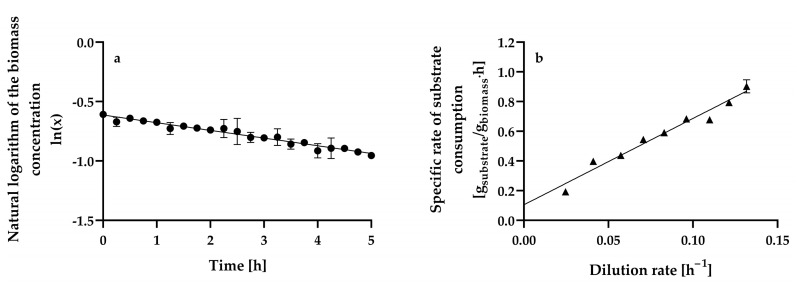
Determination of the (**a**) maximum specific growth rate (μ_max_) and (**b**) maximum growth yield (Y_max_) and maintenance energy coefficient (m) in aerobic chemostat cultures of *Hyphopichia wangnamkhiaoensis*. Data are expressed as mean values ± standard deviation from at least two biological replicates and three technical replicates (*n* ≥ 6). (●), natural logarithm of biomass concentration, (▲), specific rate of glucose consumption.

Figure 4a shows the changes in the natural logarithm of dry biomass weight L^−1^ with time in a continuous culture of *H. wangnamkhiaoensis* operated with a dilution rate (D) of 0.2039 ± 0.0008 h^−1^, in which the yeast cells were flowing out of the culture faster than they were produced (wash-out method).

The relationship between the natural logarithm of dry biomass weight L^−1^ and time is linear (Equation (1)), with a negative slope equal to ($\mu_{max}-D$) [35]:

(1) $$\ln X=\ln X_{0}+\left(\mu_{max}-D\right)t$$

where *X*_0_ is the steady-state biomass concentration at a dilution rate of 0.0961 ± 0.0004 h^−1^, *X* is the biomass concentration in the continuous culture operating at a dilution rate (D) of 0.2039 ± 0.0008 h^−1^ at time t = t h, and μ_max_ is the maximum specific growth rate (h^−1^). The μ_max_ estimated from the wash-out method was 0.1378 ± 0.0006 h^−1^, with a determination coefficient (*r*^2^) of 0.9953.

As mentioned above, the profiles of steady-state biomass concentration (Figure 2a) and residual substrate concentration in a continuous culture (Figure 2b) are consistent with the Monod model, which describes the growth rate as a function only of the growth-limiting substrate concentration and assumes no dependence on the concentration of other nutrients and no inhibition by substrate or products [49,58]. The Monod model is expressed as follows (Equation (2)):

(2) $$\mu =\mu_{max}\cdot \frac{S}{K_{s}+S}$$

where *K_s_* is the substrate affinity, half-rate saturation, or Monod constant. When Equation (2) is applied to continuous culture, it becomes (Equation (3))

(3) $$D=D_{crit}\cdot \frac{S}{K_{s}+S}$$

where *D_crit_* is the critical dilution rate. *D_crit_* is hypothetically equal to μ_max_ and represents the maximum dilution rate at which the chemostat may be operated since at dilution rates higher than the critical dilution rate ($D>D_{crit}$), complete wash-out occurs [35,59].

Therefore, the D_crit_ for *H. wangnamkhiaoensis* in continuous culture is D_crit_ = μ_max_ = 0.1378 ± 0.0006 h^−1^, which is close to the μ_max_ (0.1368 ± 0.0055 h^−1^) obtained in batch cultures of *H. wangnamkhiaoensis* [14]. This last finding agrees with studies arguing that D_crit_ usually corresponds to the maximum specific growth rate in batch cultures [35,49].

The Monod model constant (*K_s_*) was estimated from non-linear regression analysis of experimental data, obtaining a value of 0.4166 ± 0.042 g of glucose L^−1^. This value is higher than most of the K_s_ values reported for different microorganisms cultured on distinct substrates and under different environmental conditions [49,60]. K_s_ values for glucose lower than 0.15 g L^−1^ have been determined for batch and chemostat cultures of *S. cerevisiae* [49,60,61,62,63], while a value of 0.022 g L^−1^ has been reported for the aerobic chemostat culture of *S. carlsbergensis* [55]. In contrast, when *S. cerevisiae* was grown in fed-batch cultures with a glucose concentration of 100 g L^−1^, a K_s_ value of 28 g L^−1^ was determined, and this was attributed to the catabolic repression of the respiratory enzymes caused by the high glucose concentration [63].

K_s_ is considered inversely proportional to a microorganism’s affinity for its growth-limiting substrate, and consequently, the lower the K_s_ constant, the higher the affinity for the substrate [35,63]. K_s_ strongly depends on biotic and abiotic culture conditions, including pH, temperature, oxygen levels, ionic strength, culture medium composition, and substrate assimilation, among others [60,63].

The specific glucose consumption rate increased linearly over the range of dilution rates assayed (*r*^2^ = 0.9958; Figure 4b), suggesting that fermentation was limited only by the glucose concentration in the culture medium. This trend can be expressed by Equation (4) by assuming that the quantity of products formed is small [35,58]:

(4) $$q_{s}=\frac{1}{Y_{max}}\cdot D+m$$

where *q_s_* is the specific glucose consumption rate (g of glucose g^−1^ of biomass h^−1^), *Y_max_* is the maximum biomass yield, also known as the true or theoretical biomass yield (g of biomass g^−1^ of glucose), and *m* is the maintenance energy coefficient. *Y_max_* and *m* were calculated from the slope and ordinate intercept, respectively, obtaining values of 0.1720 ± 0.0069 g of biomass g^−1^ of glucose and 0.1047 ± 0.0206 g of glucose g^−1^ of biomass h^−1^, respectively.

As expected, Y_max_ (0.1720 ± 0.0069 g of biomass g^−1^ of glucose) was higher than Y (0.1371 ± 0.0165 g of biomass g^−1^ of glucose) because Y_max_ is the highest possible biomass yield, since it considers that the consumption of substrate is only for growth purposes; therefore, no substrate is consumed for cell maintenance and product formation [35,64]. The Y_max_ of *H. wangnamkhiaoensis* is lower than that reported for *B. subtilis* PRF93 (0.4611 ± 0.0110 g of biomass g^−1^ of glucose) in glucose-limited chemostat cultures [30].

Furthermore, the maintenance energy coefficient represents the amount of substrate consumed to provide the energy required to maintain cells in a viable state without leading to increased cell mass or special product synthesis, including processes such as the maintenance of chemical gradients (e.g., regulation of internal pH, osmotic work, etc.), resynthesis of cell constituents that are continuously being degraded (turnover), transport of nutrients from the surrounding medium through the cell membrane to the cytosol, DNA unwinding and repair, etc. [35,52,58,65]. The maintenance energy coefficient of glucose-limited chemostat cultures of *B. subtilis* PRF93 (0.1170 ± 0.0180 g of substrate g^−1^ of biomass h^−1^) [30] is higher than that of *H. wangnamkhiaoensis* (0.1047 ± 0.0206 g of glucose g^−1^ of biomass h^−1^). The above results indicate that *B. subtilis* consumes more glucose for biomass production and maintenance purposes than does *H. wangnamkhiaoensis*.

### 3.3. Effect of Dilution Rate on Riboflavin Production by H. wangnamkhiaoensis

**Figure 5:**
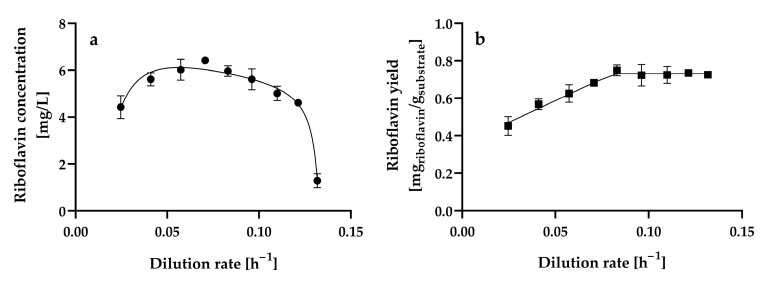
Steady-state (**a**) riboflavin concentration and (**b**) riboflavin yield by *Hyphopichia wangnamkhiaoensis* at different dilution rates in a chemostat continuous culture. Data are expressed as mean values ± standard deviation from at least two biological replicates and three technical replicates (*n* ≥ 6). (●) riboflavin concentration, (■) riboflavin yield based on glucose consumed.

Figure 5a shows the steady-state riboflavin concentration at the different dilution rates assayed.

The riboflavin concentration gradually increased from 4.42 ± 0.49 mg L^−1^ to 6.42 ± 0.02 mg L^−1^ as the dilution rate increased from 0.0246 ± 0.001 h^−1^ to 0.0706 ± 0.0004 h^−1^, and at higher dilution rates, the riboflavin concentration decreased. Therefore, the dilution rate (0.0706 ± 0.0004 h^−1^) at which maximum riboflavin production is achieved is approximately 50% of the critical dilution rate (0.1378 ± 0.0006 h^−1^) (D ≈ 0.5D_crit_). In contrast, maximum riboflavin production by *C. tropicalis* cells immobilized in calcium alginate beads occurred at a dilution rate of 0.008 h^−1^, which was the lowest dilution rate assayed, and decreased by 55 and 76% as the dilution rate increased from 0.008 h^−1^ to 0.013 h^−1^ and 0.019 h^−1^, respectively [25]. Furthermore, steady-state riboflavin concentrations lower than 1 mg L^−1^ were produced in glucose-limited chemostat continuous cultures of *A. gossypii* ATCC 10855 at dilution rates ranging from 0.1 h^−1^ to 0.32 h^−1^ [28]. Further experiments confirmed that *A. gossypii* ATCC 10855 did not produce riboflavin at constant dilution rates. However, a decline in the growth rate of the fungus caused by a downshift in the dilution rate from 0.2 to 0.05 h^−1^ during continuous cultivation resulted in riboflavin overproduction [27]. *B. subtilis* PRF93 exhibited the highest riboflavin production in glucose-limited chemostat cultures at dilution rates ranging from 0.2 to 0.45 h^−1^, with a maximum at a dilution rate of 0.3 h^−1^ (approximately 90 mg L^−1^); however, riboflavin production was highly unstable at dilution rates lower than 0.15 h^−1^ [30].

The steady-state riboflavin yields from glucose of *H. wangnamkhiaoensis* increased linearly from 0.4521 ± 0.0503 to 0.7487 ± 0.0290 mg of riboflavin g^−1^ of glucose as the dilution rate increased from 0.0246 ± 0.0010 to 0.0830 ± 0.0003 h^−1^ (Figure 5b). From this last dilution rate, the steady-state riboflavin yield remained almost constant as the dilution rate increased (Figure 5b). In contrast, in glucose-limited chemostat cultures of *B. subtilis* RB50::pRF69, the riboflavin yield dropped threefold when the dilution rate was increased from 0.03 to 0.16 h^−1^. This observation was attributed to changes in intracellular metabolism, co-metabolism of by-products accumulated during batch growth, and/or genetic degeneration and selection of a subpopulation with lower riboflavin production [32].

**Figure 6:**
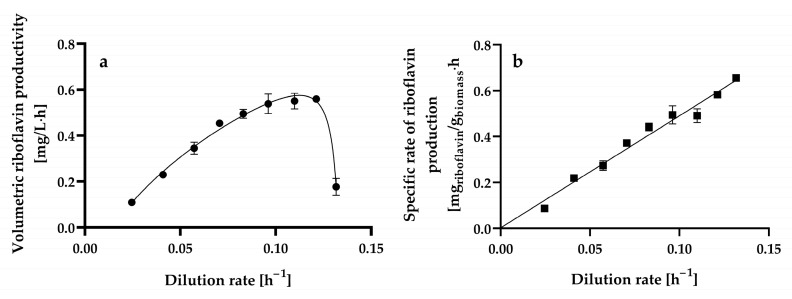
Steady-state volumetric riboflavin productivity (**a**) and specific riboflavin production rate (specific riboflavin productivity) (**b**) at different dilution rates by *Hyphopichia wangnamkhiaoensis* in chemostat continuous culture. Data are expressed as mean values ± standard deviation from at least two biological replicates and three technical replicates (*n* ≥ 6). (●), volumetric riboflavin productivity; (■), specific rate of riboflavin production.

The steady-state volumetric riboflavin productivity gradually increased up to a dilution rate of 0.1213 ± 0.0004 h^−1^ when a maximal level of 0.5593 ± 0.0016 mg L^−1^ h^−1^ was reached, as shown in Figure 6a. At higher dilution rates, the volumetric riboflavin productivity decreased significantly.

The highest volumetric riboflavin productivity achieved in chemostat continuous culture (0.5593 ± 0.0016 mg L^−1^ h^−1^) was similar to and higher than the productivities obtained in batch culture when considering the fermentation time and the entire processing time (culture turnaround, delay, and fermentation times) (0.4172 ± 0.0028 mg L^−1^ h^−1^), respectively [14].

The specific rate of riboflavin production (specific riboflavin productivity) and dilution rate exhibited a linear kinetic relationship with a positive slope and ordinate intercept (Figure 6b). This relationship resembles the one proposed for products whose formation is partially dependent on and independent of cell growth (i.e., mixed-growth-associated products), which can be expressed satisfactorily [determination coefficient (*r*^2^) = 0.994] using the Luedeking–Piret model [66] (Equation (5)):

(5) $$q_{p}=\alpha \cdot D+\beta$$

where *q_p_* is the specific riboflavin production rate (mg of riboflavin g^−1^ of biomass h^−1^), *α* is the growth-associated riboflavin production constant (mg of riboflavin g^−1^ of biomass), and *β* is the non-growth-associated riboflavin production constant (mg of riboflavin g^−1^ of biomass h^−1^).

The α and β values for riboflavin production by chemostat continuous cultures of *H. wangnamkhiaoensis* were calculated from the slope and ordinate intercept and found to be 4.8803 ± 0.2051 mg of riboflavin g^−1^ of biomass and 0.0022 ± 0.0173 mg of riboflavin g^−1^ of biomass h^−1^, respectively. These results show that α is much higher than β, implying that riboflavin production by *H. wangnamkhiaoensis* is much more strongly associated with cell growth [67,68]. Previous studies conducted in batch cultures of *H. wangnamkhiaoensis* also showed that riboflavin production is mostly associated with cell growth [14]. Furthermore, riboflavin production by glucose-limited chemostat cultures of *B. subtilis* PRF93 also followed the kinetic pattern of a mixed-growth-associated product [30].

The highest specific rate of riboflavin production achieved in chemostat continuous culture by *H. wangnamkhiaoensis* was 0.6547 ± 0.0152 mg of riboflavin g^−1^ of biomass h^−1^, which was 149% and 358% higher than those obtained in batch culture when considering the fermentation time and the entire processing time (culture turnaround, delay, and fermentation times), respectively [14]. The substantial advancement achieved in enhancing specific riboflavin productivity through steady-state continuous culture indicates promising prospects for even greater effectiveness, efficiency, and sustainability in riboflavin production through future developments.

The information obtained from the present study (kinetic parameters, mathematical models, etc.) is very useful for the optimization, modeling, and simulation of riboflavin production processes by *H. wangnamkhiaoensis*, which are currently being conducted in our laboratory. This information is also helpful for the future scaling-up of riboflavin production by *H. wangnamkhiaoensis*.

## 4. Conclusions

Chemostat continuous cultures were used to investigate the steady-state kinetics of cell growth, glucose consumption, and riboflavin production by *H. wangnamkhiaoensis* at different dilution rates. Furthermore, relevant kinetic parameters describing yeast growth, glucose consumption, and riboflavin production in continuous cultures were also determined. The Monod model described yeast cell growth satisfactorily, while the Luedeking–Piret model represented riboflavin production well. Riboflavin production by *H. wangnamkhiaoensis* was a predominantly growth-associated process. Specific riboflavin productivity was significantly enhanced in continuous cultures compared with that obtained in batch cultures, demonstrating the potential of chemostat fermentation for effective and sustainable riboflavin production by *H. wangnamkhiaoensis*.

## Data Availability

All relevant data are within the paper.
